# Neurotransmitter uptake of synaptic vesicles studied by X-ray diffraction

**DOI:** 10.1007/s00249-022-01609-w

**Published:** 2022-07-29

**Authors:** Karlo Komorowski, Julia Preobraschenski, Marcelo Ganzella, Jette Alfken, Charlotte Neuhaus, Reinhard Jahn, Tim Salditt

**Affiliations:** 1 Institute for X-ray Physics, University of Göttingen, Friedrich-Hund-Platz 1, 37077 Göttingen, Germany; 2 Department of Neurobiology, Max Planck Institute for Biophysical Chemistry, Am Faßberg 11, 37077 Göttingen, Germany

**Keywords:** Synaptic vesicles, Small angle X-ray scattering, Neurotransmitter uptake, Synchrotron and free electron laser techniques

## Abstract

The size, polydispersity, and electron density profile of synaptic vesicles (SVs) can be studied by small-angle X-ray scattering (SAXS), i.e. by X-ray diffraction from purified SV suspensions in solution. Here we show that size and shape transformations, as they appear in the functional context of these important synaptic organelles, can also be monitored by SAXS. In particular, we have investigated the active uptake of neurotransmitters, and find a mean vesicle radius increase of about 12% after the uptake of glutamate, which indicates an unusually large extensibility of the vesicle surface, likely to be accompanied by conformational changes of membrane proteins and rearrangements of the bilayer. Changes in the electron density profile (EDP) give first indications for such a rearrangement. Details of the protein structure are screened, however, by SVs polydispersity. To overcome the limitations of large ensemble averages and heterogeneous structures, we therefore propose serial X-ray diffraction by single free electron laser pulses. Using simulated data for realistic parameters, we show that this is in principle feasible, and that even spatial distances between vesicle proteins could be assessed by this approach.

## Introduction

**Fig. 1:**
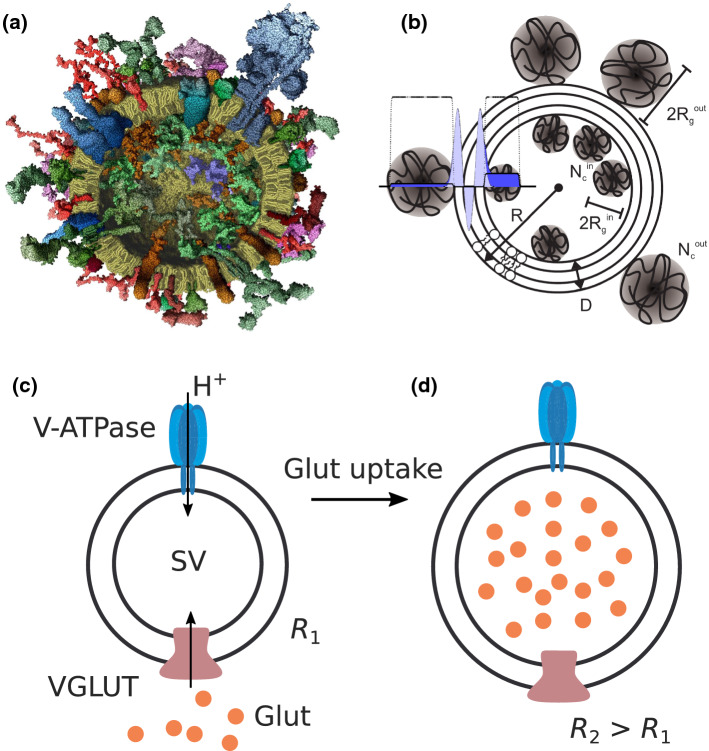
Structure and function of SVs. **a** Molecular model of an average SV based on biochemical knowledge, adapted from (Takamori 2006). **b** Sketch of the anisotropic SAXS model. The radial electron density profile (EDP) of the lipid bilayer is modeled by the sum of three Gaussians (one Gaussian for each headgroup region and one Gaussian for the hydrophobic chain region). The inner and outer protein layers are modeled as Gaussian chains. **c** Illustration of glutamate (Glut) uptake by SVs. Glutamate is loaded by the vesicular glutamate transporter (VGLUT) into SVs. The driving energy for neurotransmitter uptake is provided by an electrochemical gradient established by a vacuolar-type ATPase (V-ATPase), which translocates protons ($\mathrm {H}^{+}$) into the vesicle interior using the energy derived from ATP hydrolysis

Neurotransmission at chemical synapses relies on synaptic vesicles (SVs) as highly specialized small organelles containing neurotransmitters. Triggered by an influx of $\mathrm {Ca}^{2+}$ during neuronal stimulation, SVs fuse with the plasma membrane (exocytosis), release their neurotransmitter content into the synaptic cleft, and are recovered again by endocytosis only to be refilled with neurotransmitter for the next round of exocytosis (Südhof 2004; Jahn and Fasshauer 2012). A comprehensive molecular model integrating all quantitative data on the protein and lipid composition of the SV has been presented in Takamori (2006), and is shown in Fig. 1a. SVs and in particular the SNARE (soluble *N*-ethylmaleimide-sensitive-factor attachment receptor) protein machinery regulating SV fusion have been intensively studied (Jahn and Scheller 2006; Hernandez et al. 2012; Kliesch et al. 2017). While molecular composition of SVs can be well analyzed by various biochemical techniques, and the structure of its protein constituents by structural biology techniques (Sutton et al. 1998), structural details at level of the organelle level, in particular regarding the arrangement of proteins and lipids are difficult to obtain directly from microscopy techniques. Given the small SV radius around $R\simeq 19$ nm, imaging of SVs by fluorescence microscopy requires super-resolution techniques (Willig et al. 2006) or electron microscopy (EM) of cryogenically vitrified sections (Takamori 2006), in which inner and outer protein layers can be discerned, however, without much structural details. Using small-angle X-ray scattering (SAXS), our group has investigated the size and structure of purified SVs directly in solution (Castorph et al. 2010a). Based on a scattering model (Castorph et al. 2010a, b), which is illustrated in Fig. 1b, we could deduce detailed size and density parameters for the protein layers, as well as structural information about the lipid bilayer. A laterally anisotropic structure for the protein shell, indicative for protein microdomains yielded very satisfactory least-square fits of the measured SAXS, while a rotationally symmetric density profile fitted the data less well (Castorph et al. 2010a).

A prerequisite for analysis, both chemical and structural, is the purification of SVs in sufficient quantity. Even measurements of size as the presumingly most basic structural parameter, can in fact be already quite challenging for biological vesicles (Rupert et al. 2017; Varga et al. 2020; Perissinotto 2020). For SVs, measurements are facilitated by the large SV abundance in brain tissue and the relative size and shape homogeneity. Compared to most other vesicles and organelles, the polydispersity of SVs is relatively small, but not small enough to enable cryo-EM single particle reconstructions, which requires monodisperse particles. SAXS analysis is also significantly affected by polydispersity, as discussed further below in this work. For this reason, SAXS is also very well suited to validate purification protocols. However, at some point the intrinsic heterogeneity associated with the physiological processes of SV formation and recycling, as well as the corresponding variations in copy numbers will set a limit to most analysis techniques. This may come as a nuisance, but also reflects an important physiological fact which can shed light on the robustness of functional processes. As active energy-driven processes, uptake, fusion, release and recycling require tight temporal and spatial control, and beyond the basic ‘anatomy’ of SVs, the next challenge is to shed more light on these processes and their structural dynamics. A particular case in point is the neurotransmitter uptake based on active pumping, for example by the vesicular glutamate transporter 1 (VGLUT1) energized by V-ATPase, as sketched in Fig. 1c. Much is known already on the regulation of VGLUT1 and other vesicular neurotransmitter transporter (Ahnert-Hilger et al. 2004), as well as on the relationship between neurotransmitter transport activity and vesicle filling (Edwards 2007). The transport is driven by the V-ATPase-dependent electrochemical proton gradient ($\Delta \mu H^+$) and can be stimulated by low concentration of $\mathrm {Cl}^{-}$. Using a reconstitution approach, it was shown that VGLUT1 contains two anion binding sites and one cation binding site, allowing the transporter to adjust to the changing ionic conditions during vesicle filling without being dependent on other transporters or channels (Preobraschenski et al. 2014). In addition to glutamate transport, VGLUT1 can also perform bidirectional phosphate transport and may play a role in neuronal phosphate homeostasis (Preobraschenski et al. 2018).

In this work, we use SAXS combined with active SV preparations to study the size increase associated with glutamate uptake. The starting point of our investigation was the surprisingly large increase in SV radius *R* after glutamate uptake, up to $25\%$, reported in Budzinski et al. (2009), corresponding to about a doubling of the volume! The effect was first observed by fluorescence correlation spectroscopy (FCS). To rule out that this was only an apparent size increase resulting from changes in the hydrodynamic radius or the diffusion properties, the authors verified the result by cryo-EM. The authors did not observe the size increase if SV-specific proteins notably SV2A (synaptic vesicle glycoprotein 2A) were absent, and discussed different models accounting for the vesicle expansion. To this end, they distinguished three different mechanisms: (i) an island model with the vesicle surface composed of non-expandable lipids and of a second phase composed of expandable protein components, (ii) a virus-like model exhibiting expandable breathing modes, and (iii) a matrix-swelling model where a gel formed by SV2A sugar moieties binds the glutamate even if the membrane ruptures upon expansion. The first goal of this work is to verify the size increase by using solution SAXS. Note that while FCS probes structural changes only indirectly via diffusion properties, cryo-EM gives direct access to the size of individual particles, but only in the vitrified state. Further cryo-EM is limited to a relatively small number of particles which can be probed. Contrarily, SAXS probes the average structure of a large ensemble, but in contrast to a direct imaging method such as cryo-EM requires least-square fitting to a parameterized model, see for example (Székely et al. 2010). The advantage here is the fact that potentially more structural parameters can be extracted, in particular a parameterized density profile with more details on the structural rearrangements for example in the inner and outer SV protein layers. As we show here, SAXS can confirm the size increase, albeit by a factor of about $\Delta R/R \simeq {0.12}$, hence about a factor of two smaller than the largest values reported in Budzinski et al. (2009). Further, we obtain some indications for significant protein rearrangements (possibly conformational changes) in the protein layers. With our earlier study of equilibrium SVs suspensions as a SAXS benchmark (Castorph et al. 2010a), we now ask about the potential and limitations of solution SAXS concerning functional dynamics and out-of-equilibrium processes. As we show here, while some insight and additional information can be derived from uptake studies, the level of details which can be robustly derived from the model fits is largely limited by the intrinsic polydispersity of the SV suspension. As a solution to this problem, we propose high throughput single particle coherent diffractive imaging with femtosecond X-ray free electron laser (XFEL) pulses in the outlook of this paper, and corroborate feasibility by numerical simulations.

The manuscript is organized as follows: after this introduction, the methods section details sample preparation, SAXS measurements, and also gives a brief recapitulation of the SAXS model and data analysis. In the results section we then first consider polydispersity and the improvements in purification and correspondingly homogeneity, before we address structural changes associated with SV functions. Aligned with the main goal of this work, we present the SAXS results for SVs after glutamate uptake, and quantify the associated size increase. After the results section, we discuss the limiting effects of polydispersity in deducing structural parameters from scattering, and close with an outlook proposing an alternative approach based on sequential single-pulse coherent diffraction with XFEL radiation, which we substantiate with numerical simulations.

## Materials and methods

### Preparation of the uptake experiment

Synaptic vesicles were purified from rat brain, as described in Takamori (2006), Kreutzberger et al. (2019). After purification, SV samples were frozen and stored at $-80$ $^{\circ }\mathrm {C}$. Further sample preparation was performed directly before the SAXS experiments. First, the SVs were thawed on ice for approximately 15 min. For buffer exchange, the SVs were dialysed against the buffer consisting of 300 mM glycine, 10 mM KCl, 5 mM HEPES, 2 mM $\mathrm {MgSO}_{4} \times 7 \mathrm {H}_{2}\mathrm {O}$ (pH 7.3) at 4 $^{\circ }\mathrm {C}$ for approximately 3 h. To this end, the SV samples were injected into Slide-A-Lyzer cassettes (Thermo Fisher Scientific, Waltham, MA) with a molecular weight cutoff of 2 kDa. For the uptake experiment and for the control experiment, 10 mM K-glutamate and 1 mM Mg-ATP, and 10 mM K-glutamate, respectively, was added to the SV sample and incubated in a thermo-mixer at 37 $^{\circ } \mathrm {C}$ for 10–15 min just before the samples were injected into the sample chamber for the SAXS experiments.

### Small-angle X-ray scattering

#### SAXS measurements

SAXS experiments were performed at the undulator beamline ID02 (Narayanan et al. 2018) at European Synchrotron Radiation Facility (ESRF) in Grenoble, France. The beamline was operated at 12.45 keV photon energy. The beam size at the sample-plane was $100 \times 100$ $\mu$m. The samples were measured at two sample-to-detector distances, 1.5 and 5 m, to cover a *q*-range of approximately 0.02–3.37 $\mathrm {nm}^{-1}$ after merging the SAXS signals. The scattered X-rays were recorded by a Rayonix MX-170HSCCD pixel detector (Rayonix L.L.C., USA) with $3840 \times 3840$ pixels. The two-dimensional isotropic diffraction pattern was calibrated to the absolute scale (water reference). For the SAXS measurements, the samples were loaded into a flow-through capillary cell (1.6 mm in diameter). The sample chamber was heated to 37 $^{\circ }\mathrm {C}$. For each measurement, 10 SAXS signals were recorded with an exposure time of 1 s, and averaged after azimuthal integration. The SAXS signals obtained at the two detector distances were then merged. For background subtraction, the matched buffer was measured separately.

#### SAXS analysis

For completeness and notational clarity we include a brief recapitulation of the SAXS analysis for SVs, as developed in Castorph et al. (2010a, 2010b), and summarized also in Salditt et al. (2019). The incident X-ray beam with wave vector $\mathbf {k}_{i}$ and wave number $|\mathbf {k}| = 2 \pi / \lambda$ for wavelength $\lambda$ is scattered from an isotropic suspension of SVs. The scattered X-rays with wave vector $\mathbf {k}_{j}$ and momentum transfer $\mathbf {q} = \mathbf {k}_{j} - \mathbf {k}_{i}$ are recorded on the 2*d* area detector. The isotropic diffraction pattern depends only on the scattering angle $2\theta$, or correspondingly the modulus of the momentum transfer $q = |\mathbf {q}| = 4 \pi / \lambda ~ \text {sin} ~ \theta$. The intensity for a dilute, polydisperse system of particles of radius *R* with the number size distribution *p*(*R*) follows from an incoherent polydispersity integration, is modelled by

1 $$\begin{aligned} I_\mathrm{mod}(q) = \Delta \rho ^{2} \int \limits _{0}^{\infty } p(R) V_{\mathrm {p}}(R)^{2} F(q,R) \mathrm {d}R, \end{aligned}$$

where $\Delta \rho$ is the average electron density contrast between the solvent and the particle, $V_{\mathrm {p}}(R)$ is the volume of the particle. Here this volume corresponds to the total volume of the SV minus the volume of the vesicle lumen (core). The relationship between scattering curve and particle structure is contained in the form factor $F(q,R) = \langle |f(\mathbf {q},R)|^{2} \rangle$ with the form factor amplitude $f(\mathbf {q},R)$ and $\langle ... \rangle$ denoting the powder average. For *p*(*r*), we used a bimodal size distribution composed of two Gaussian distributions accounting for size distribution for the SVs, as well as for larger membranous particles in the sample.

The SAXS model for SVs is schematically illustrated in Fig.1. The radial electron density profile (EDP) $\rho (r)$ for the lipid bilayer is modeled by three Gaussians, which also includes contributions of transmembrane proteins and amino acid residues associated with the headgroups. Further, the proteins of the inner and outer protein shell are modeled as Gaussian chains. It is important to note that the Gaussian chains, which break the spherical symmetry, are proxies for distinct protein patches. They are characterized by an effective radius of gyration $R_{\mathrm {g}}$ and an effective copy number of protein patches $N_{\mathrm {c}}$. The scattering length density profile of the lipid bilayer with partial and symmetrized protein contributions is accounted for

2 $$\begin{aligned} \rho (r) = \sum _{i}{\rho _i \exp \left( -\frac{(r-R_i)^2}{2 t_i^2}\right) } ~, \end{aligned}$$

where $R_i$ is the peak position, $\rho _i$ is the amplitude and $t_i$, $i \in \{in,out,tail\}$ is the width, for each of the Gaussians representing the headgroups and the tail region. The thickness of the bilayer is defined as $D = \sqrt{2\pi }(t_{in} + t_{tail} + t_{out})$, where $t_{in} = t_{out}$ is chosen to describe a symmetric bilayer. In this work, the vesicle radius *R* is defined by the center of the bilayer, i.e. by $R_{tail}$, in contrast to Castorph et al. (2010a), where it was defined as the outer bilayer surface $R=R_{out} + t_{out} \sqrt{2\pi }/2$. The total excess scattering length of the bilayer with respect to the aqueous buffer is $\beta _\mathrm{b}$. The Gaussian chains are distributed randomly and without correlations forming the inner and outer protein shell with effective copy numbers $N_{c}^{in}$ and $N_{c}^{out}$, respectively. They are further characterized by their radii of gyration, $R_{g}^{in}$ and $R_{g}^{out}$, and by their average excess scattering length density $\rho _c$. The distance between the inner headgroup and the center of mass of the Gaussian chains facing the lumen is $t_{in} \sqrt{2\pi }/2 + R_{g}^{in}$, and the distance between the outer headgroup and the center of mass of the Gaussian chains facing outwards is $t_{out} \sqrt{2\pi }/2 + R_{g}^{out}$. In this way, the Gaussian chains partly overlap with the tails of the bilayer profile, but do not fully penetrate the bilayer. The combination of these results leads to the following form factor

3 $$\begin{aligned} \nonumber F(q,R)= & {} \dfrac{1}{M^{2}} \times [\beta _b^2 F_{b}^2(q,R)\nonumber \\&+ \sum _{i=in ,out}^{}{N_{c}^{i} \beta _{c}^{i \ 2} P_{c}^{i}(q)}\nonumber \\&+ \sum _{i=in ,out}^{}{2N_{c}^{i \ 2} \beta _b \beta _{c}^{i} S_{b \ c}^{i}(q,R)}\nonumber \\&+ \sum _{i=in ,out}^{}{N_{c}^{i}(N_{c}^{i}-1) \beta _{c}^{i \ 2} S_{c}^{i}(q,R)} \nonumber \\&+ {~S_{c}^{in \ out}(q,R)} \prod _{i=in ,out}^{}{N_{c}^{i} \beta _{c}^{i}} ~]. \end{aligned}$$

The different terms are now described in the following. $M = \beta _b + N_{c}^{in} \beta _{c}^{in} + N_{c}^{out} \beta _{c}^{out}$ denotes the excess scattering length, with $\beta _{c}^{i} = \frac{4\pi }{3} {R_g^i}^3 \rho _c$ the total excess scattering length of a single Gaussian chain in the modeled protein layer and with $i=in, out$, as in all following equations. The first term contains the normalized amplitude of the self-correlation of the bilayer profile, given by

4 $$\begin{aligned} F_{b}(q,R) = \sum _{i=in ,tail ,out}^{}{\dfrac{F_{b \ i}(q,R_i)}{M_{b\ i}}} + F_{\mathrm {lumen}}, \end{aligned}$$

with

5 $$\begin{aligned} F_{b \ i}(q,R_i)= & {} 4 \sqrt{2} t_i \rho _i \exp \left( - \frac{t_i^2 q^2 }{2}\right) ~q^{-1} \nonumber \\&~[t_i^2 q \cos (qR_i) + R_i \sin (q R_i) ] ~, \end{aligned}$$

and $F_{\mathrm {lumen}}=\rho _{\mathrm {lumen}} V (\sin qR_v - qR_v \cos (q R_v))/(q R_v)^3$ the form factor of the vesicle lumen modeled as an ideal sphere with radius $R_v=R-(t_{in}+t_{tail}/2)\sqrt{2\pi }$ and the excess scattering length density $\rho _{\mathrm {lumen}}$ (i.e. the density contrast to the buffer) to account for changes in density due to neurotransmitter uptake. Note that in the original model $\rho _{\mathrm {lumen}}=0$ (Castorph et al. 2010a), since in that work only inactive SVs were considered. $M_{b \ i} = \rho _i \frac{4 \pi }{3} ((R_i + t_i \sqrt{2\pi }/2)^3-(R_i - t_i \sqrt{2\pi }/2)^3)$ is the excess scattering length of one peak of the bilayer profile. The second term in the form factor describes the self-correlation terms of the Gaussian chains:

6 $$\begin{aligned} P_{c}^{i}(q) = \dfrac{2[\exp (-x^i) - 1 + x^i]}{x^{i \ 2}} ~, \end{aligned}$$

with $x^i = q^2 R_{g}^{i \ 2}$. The third term accounts for the interference cross-terms $S_{b \ c}^{in}(q,R)$ and $S_{b \ c}^{out}(q,R)$ between the bilayer and the Gaussian chains, given by

7 $$\begin{aligned} S_{b \ c}^{i}(q,R) = F_b(q,R) \psi ^{i}(x^{i}) \dfrac{\sin (q[R_{tail} \mp (D/2 + R_g^i)])}{q[R_{tail} \mp (D/2 + R_g^i)]} ~, \end{aligned}$$

where $\psi ^i(x^i) = [1 - \exp (-x^i)]/x^i$ the effective form factor amplitude of the Gaussian chains. Finally, the fourth term describes the interference of chains inside and outside the bilayer

8 $$\begin{aligned} S_{c}^{i}(q,R) = \left[ \psi ^{i}(x^{i}) \dfrac{\sin (q[R_{tail} \mp (D/2 + R_g^i)])}{q[R_{tail} \mp (D/2 + R_g^i)]}\right] ^2, \end{aligned}$$

and the interference between the chains of the inner and outer shells across the bilayer is taken into account by the fifth term

9 $$\begin{aligned} S_{c }^{in \ out}(q,R)= \prod _{i=in ,out}^{}{ \psi ^{i}(x^{i}) \dfrac{\sin (q[R_{tail} \mp (D/2 + R_g^i)])}{q[R_{tail} \mp (D/2 + R_g^i)]}} ~. \end{aligned}$$

**Least-squares fit.** To obtain structural parameters from SAXS data, the experimental scattering intensities $I_{\text {exp}}(q_{i})$ with data points $i=1,...,N$ recorded at $q_{i}$, were fitted by the model curve $I_{\text {mod}}(q_{i})$, accounting for a scaling factor and a constant background as

10 $$\begin{aligned} I_{\text {exp}}(q) = c_{1} \cdot I_{\text {mod}}(q) + c_{2} ~. \end{aligned}$$

The quality of the fit was monitored by the reduced $\chi ^{2}$-function

11 $$\begin{aligned} \chi _{\text {red}}^{2} = \frac{\sum \nolimits _{i=1}^{N} \frac{[I_{\text {exp}} (q_{i})-I_{\text {tot}}(q_{i})]^{2}}{\sigma _{i}^{2}}}{N-p-1} ~, \end{aligned}$$

where *p* is the number of free model parameters and $\sigma _{i}^{2}$ is the variance of the intensity $I_{\text {exp}}(q_{i})$ for a measured data point *i*. Nonlinear least-squares fitting was implemented using the MATLAB function *lsqnonlin* of the MATLAB R2020b Optimization Toolbox. Since the estimation of partial derivatives from the fitting routine was unstable, the fitting errors and covariance matrix of the parameters was determined as follows: Random pseudo-realizations of ‘experimental’ data points were drawn from a normal distribution centered around the true experimental data points with a standard deviation given by the experimental errors (determined from error propagation of the SAXS intensities at ID02). For each *q*-values these realisations were independently generated and hundred such curves were then fitted to the same model, yielding parameter vectors form which the fit errors $\sigma _p$ for each parameter and the covariance matrix was computed.

## Results and discussion

### Polydispersity and purity

Notwithstanding well established protocols and almost two decades of experience (Takamori 2006; Kreutzberger et al. 2019), reproducible and contamination-free SV preparations extracted and purified from rat brain is always a primary concern. This is accentuated by the rather large quantities required for scattering experiments. More generally, at the organelle level, almost any fractionation and purification from higher animals is quite challenging. At the same time, the purity of the preparation is crucial for structural studies by ensemble techniques such as scattering or spectroscopy. In scattering, whether with light, X-rays or neutrons, large particle contaminations have particular strong weight in the signal, since the signal scales with the squared volume, i.e. $R^6$ for the model case of solid spheres. As a consequence, a small fraction of larger aggregates caused by contamination (aggregated or fused SVs, ruptured membrane debris, etc.), compared to the size of SVs ($R \approx 19 ~ \mathrm {nm}$), has a considerable impact on the scattering curve. In fact, the SAXS data measured by Castorph et al. could only be modeled by including an additional size distribution accounting for contamination (Castorph et al. 2010a). Large membranous particles, i.e. contamination, were also observed by cryo-EM (Castorph et al. 2010a), but can of course be vetoed out in direct microscopic observations, while they remain always in the scattering volume (cuvette) of ensemble techniques. At the same time, scattering is therefore very sensitive to detect contaminations, even if orders of magnitude smaller than the main fraction. The first point of this study therefore concerned the reproducibility of our previous results measured a decade ago (Castorph et al. 2010a), and whether there was any improvement in the level of contaminations due to refinements of the preparation protocol. To this end, we first show and compare the SAXS data of SVs measured during this study to the SAXS data published a decade ago by Castorph et al. (2010a).

**Fig. 2:**
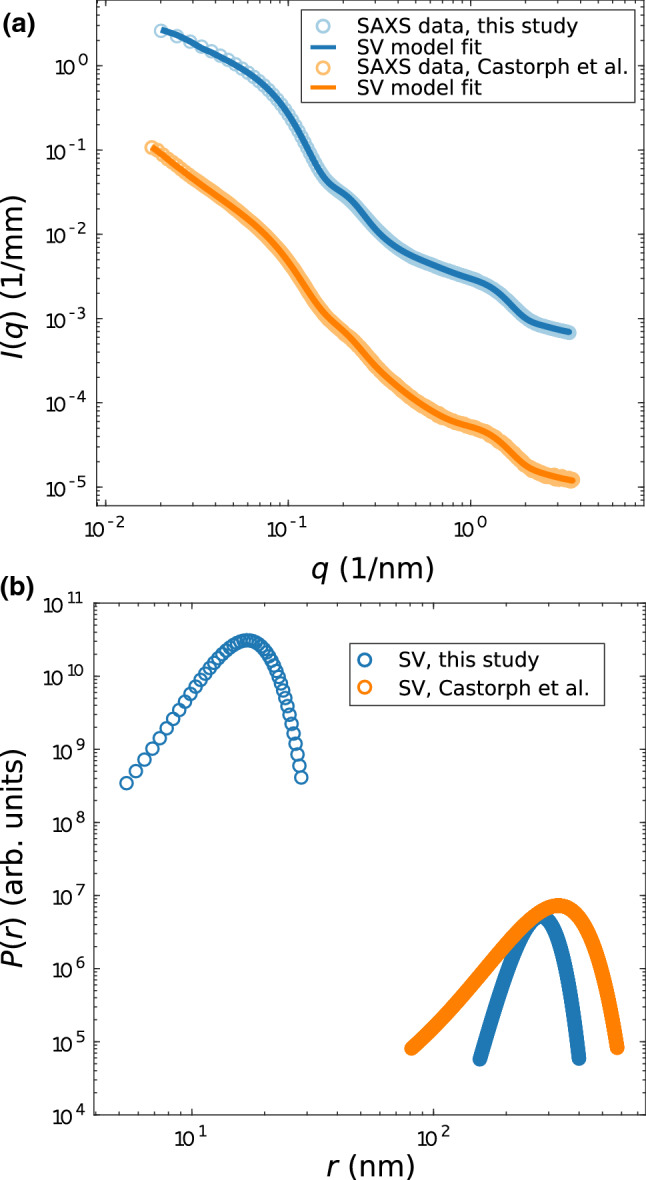
**a** Comparison of SAXS data *I*(*q*) vs. *q* obtained from (blue) SVs measured during this study and (orange) from SVs published a decade ago by Castorph et al. (2010a), and least-squares fits (SAXS data, this study: $\chi _{\mathrm {red}}^{2} = 67.8$, and SAXS data S. Castorph et al.: $\chi _{\mathrm {red}}^{2} = 4.99$). **b** Bimodal Gaussian size distributions obtained from the fits shown in **a** for SVs (this study) and SVs (S. Castorph et al.), accounting for the actual SV size distribution, and for contamination (for example, large membranous particles). The size distribution of the SV fraction was modeled as a Gaussian, fitted to the new SAXS data, and then kept constant in the fit of the Castorph et al. data. For this reason of enforced equality, **b** shows only a single color (blue) for this fraction. Contrarily, the contamination fraction was fitted freely in both datasets (see the corresponding curves in the two respective colors). The results show that the size distribution of the contamination is larger for the ’old’ SV purification (S. Castorph et al.), as compared to the purification used in this study. Fitting parameters are tabulated in Table 1

Figure 2 presents the comparison of the SV data and the analysis of contamination. By inspection of the scattering curves shown in (a), and already before any fitting, we can directly recognize the characteristic modulations of the SAXS curve in both the old and the new data sets indicating qualitative reproducibility. At the same time small differences can be observed in the functional shape, in particular for small *q*, where the more shallow slope for the new SV data indicates a smaller contribution from larger aggregates, i.e. a cleaner purification. This observation is quantified by least-squares fits using the anisotropic SAXS model for both data sets. The fit of the SV data measured during this study was parameterized as follows. The amplitudes of the EDP as well as the scale were kept constant, while all other parameters were free to vary. In other words, the protein and lipid headgroup and tail electron density was fixed at literature reference values (see Table 1), but the protein number density and radius of gyration was free. For the least-squares fit of the SAXS data from Castorph et al. (2010a), the amplitudes of the EDP and the small size distribution was kept constant, but the other structural parameters could vary freely and independently. All fit results are listed in Table 1. As a result we see that the EDP and the small size distribution, i.e. the main fraction of the SVs is well reproduced, while the amplitude of the large size distribution, i.e. the contaminations, have been reduced in the new data. The resulting size distributions are plotted in Fig. 2b. The large size distribution for the SAXS data from Castorph et al. shows a higher fraction as compared to the SV sample measured during this study, i.e. higher amplitude. Please also note the double-logarithmic scale and the fact that in the new data set, a suppression by four orders of magnitude is achieved for the large size fraction, underlining the quality of the preparation. Interestingly, the $\chi _{\mathrm {red}}^2$-values of the SAXS data from Castorph et al. is substantially lower, which may be attributed to the fact that larger polydispersity screens some of the systematic errors of the model. In other words, the discrepancy of the still overly simplistic SV SAXS model becomes more apparent for the high quality preparation. Note that compared to inhouse or second generation SAXS instruments, the higher brilliance of the ID02 undulator beamline results in very small statistical errors of the SAXS data points, and hence $\chi _{\mathrm {red}}^2 \simeq {\mathcal {O}}(1)$ is much more difficult to reach.

### Structure of SVs upon glutamate uptake

**Fig. 3:**
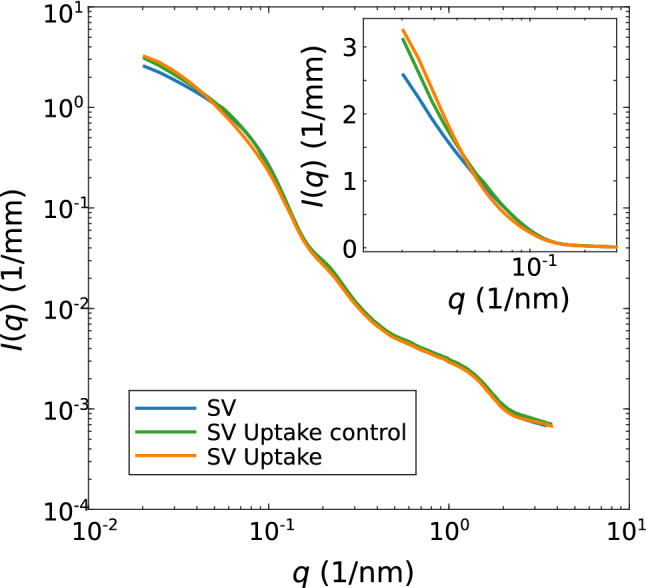
Comparison of SAXS data *I*(*q*) vs. *q* obtained from (blue) inactive SVs and (orange) active SVs upon the addition of 1 mM ATP and 10 mM K-glutamate. For the control experiment, 10 mM K-glutamate, without ATP, was added to the SV suspension (green curve). The inset shows the SAXS data set for lower *q*-values, where main differences can be observed between the different SAXS signals

Next, we have investigated the structure of SVs upon the uptake of K-glutamate. Figure 3 shows the superimposed SAXS curves obtained from (blue) SVs without glutamate and ATP, (green) SVs with added glutamate but without ATP as a control experiment, and (orange) SVs with added glutamate and ATP as the uptake experiment. The curves differ in particular for low-*q* values, showing an increase in $I(q \rightarrow 0)$ for the uptake and the control experiment with respect to the SV reference without added metabolites. Note that in this low-*q* values, the ATP-driven glutamate transmembrane transport and ATP-devoid ‘control’ curves both show an intensity increase with respect to the SV reference, but the effect is more pronounced for the active system than for the control. Importantly, the SAXS curve of the active system differs from the control over the entire q-range, by a significant amount, with respect to the statistical errors. Before turning to the more complicated least-square fit of the data to the full SV model, one may be tempted to first validate a size increase by a simple Guinier fit. However, the large particles which dominate the low-*q* range and spoil such attempts, since the size ranges do not separate.

**Fig. 4:**
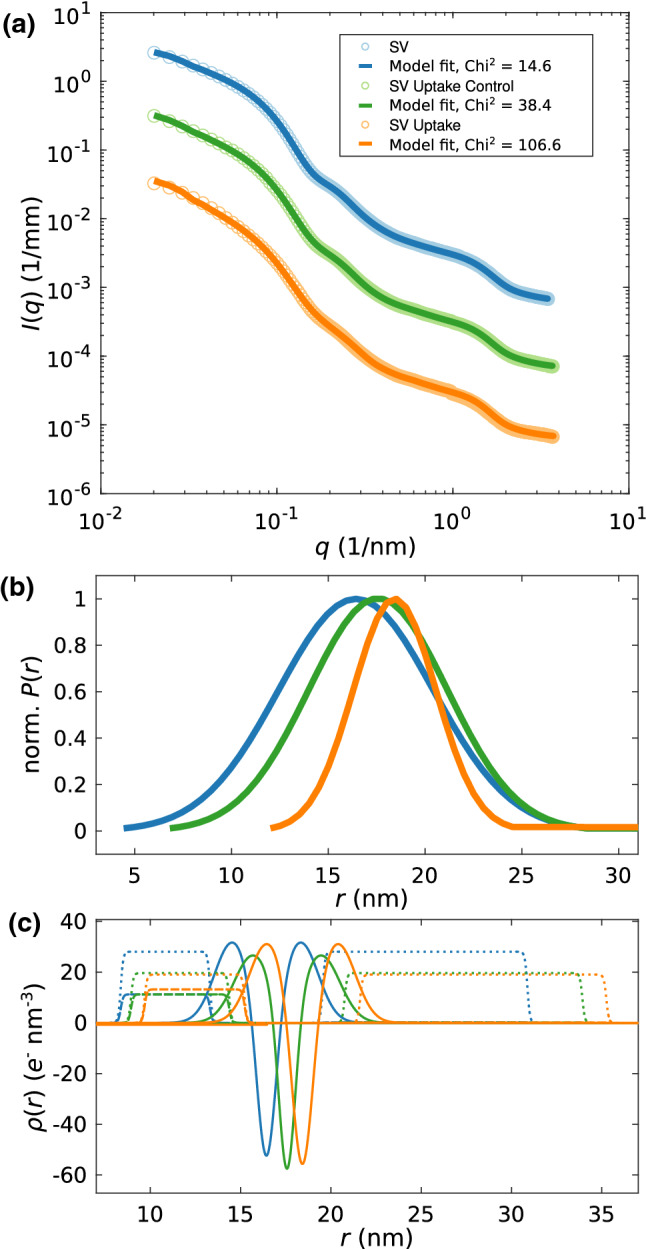
Structural changes of SVs after neurotransmitter uptake. **a** SAXS data and anisotropic-SV model fits for SV only (blue, $\chi _{\mathrm {red}}^{2} = 14.6$), SV uptake control (green, $\chi _{\mathrm {red}}^{2} = 38.4$), and SV uptake (orange, $\chi _{\mathrm {red}}^{2} = 106.6$). **b** Normalized Gaussian size distributions obtained from least-square fits shown in (**a**) with the corresponding colors. **c** Electron density profiles obtained from the least-squares fits with the corresponding colors. (Solid line) EDP of the lipid bilayer, (dotted line) Gaussian chains local, and (dashed line) Gaussian chains spherically averaged. The electron density of the inner lumen differs only slightly from zero for each EDP. For details, the fitting parameters are tabulated in Table 2

The SAXS data of the uptake experiment was therefore fitted with the anisotropic SV-SAXS model, see Fig. 4, taking into account both size fractions, as explained above. The data and the least-squares fits (solid curves) are presented in Fig. 4a, shifted vertically for clarity, for (blue) SVs, (green) SV uptake control, and (orange) SV uptake. All resulting fit parameters are tabulated in Table 2. The parameters were freely and independently varied for all three cases, including the now added parameter $\rho _{\mathrm {lumen}}$ to account for the uptake effect (see above). The fits resulted in satisfactory $\chi _{\mathrm {red}}^{2}$-values, of $\chi _{\mathrm {red}}^{2} = 14.6, 38.4, 106.6$ for SVs, SVs uptake control, and SVs uptake, respectively. Figure 4b shows the resulting size distributions (normalized to 1). An increase is observed in the mean radius *R* for the uptake experiment ($R = 18.42$ nm) compared to the inactive SVs ($R = 16.42$ nm), and the control experiment ($R = 17.56$ nm). Note that the relative error of the least-square fits is $0.5\%$ as determined by a Monte–Carlo approach where *N* related datasets are generated based on independent realizations with a statistic given by the experimental errors for each data point. Importantly, these statistical errors are smaller than the differences between the three experiments. Note as well, that by our definition, *R* refers to the bilayer center, and hence is smaller by half the bilayer thickness *D*, when one compares to cryo-EM data such as in Castorph et al. (2010a). At the same time, the width of the size distribution shows a slight decrease both for the control and the uptake experiment. More importantly, the corresponding EDPs which are displayed in (c) for (blue) SVs, (green) SV uptake control, and (orange) SV uptake, show a significant rearrangement of protein and lipid moieties. This may be taken at an indication of significant conformational changes of SV proteins which are plausible given the high level of vesicle expansion. The increase in the thickness of the outer protein layer, the decrease in local protein density (both inner and outer layer) and the decrease in the central bilayer density are the most prominent changes in the EDP. Interestingly, the local protein density decreases (dotted lines) while the average density (dashed lines) of the inner protein shell increases. This suggests a more uniform coverage of the inner vesicle monolayer with protein moieties accompanied with the uptake and vesicle swelling. Of course, the changes in the EDPs have to be regarded with caution with respect to possible over-parameterisation. Alternative fitting strategies with fixed EDPs, hence ignoring possible rearrangements in the bilayer or protein layer, are included as supporting material. They result in substantially higher $\chi _{\mathrm {red}}^{2}$, but confirm the main finding of the SV size increase to be a robust result. Notably, the fitted values of the mean radius are very similar to the results shown in Fig. 4. Interestingly, the parameter $\rho _{\mathrm {lumen}}$ which denotes the density difference of the lumen with respect to the the buffer solution, is negative in sign, but very small. This is not unlikely, since the density of glutamate solutions of molality around 0.1 mol/kg differs from water by less than $1\%$ at 37 $^\circ$C (Sembira-Nahum et al. 2008). Note that no literature values are available for the higher molality encountered here for SVs with estimated 8000 glutamate molecules per vesicle (Wang etal 2019). Finally, in order to further corroborate the robustness of the observed size increase with respect to alternative fitting strategies/models, the supporting material includes an approach where the large fraction is also freely varied as well as an isotropic fitting model composed of concentric shells.

## Conclusion and outlook

As we have seen above, the increase in vesicle radius upon uptake of neurotransmitter reported in Budzinski et al. (2009) can be qualitatively confirmed by SAXS. With about 12% relative increase in radius, however, the expansion was not quite as large as the maximum value observed in Budzinski et al. (2009). This may very well be attributed to the fact that the dense suspension of SVs used here may require still higher ATP concentration than 1 mmol/l. In other words, it may be possible that the uptake experiment here was ATP limited. For a free EDP fit, there is some indication of significant rearrangement in the protein layers in agreement with what one would expect from the two first models proposed in Budzinski et al. (2009). The electron density of the glutamate loaded vesicle lumen was found to be very close but slightly lower than that of pure buffer. From the simulation and dependencies of the SAXS curve on the different parameters, we can also conclude that polydispersity is the main limiting factor for SAXS analysis of SVs. This also screens details on the conformational changes in the protein shells.

In order to unlock the potential of diffraction for SV structural studies in a more complex functional context, we essentially have three different options: Firstly, improvement of purification and size fractionation: this may be difficult since we are most probably facing the intrinsic poydispersity of SVs, rather than an effect of non-ideal preparation. Secondly, we may increase data diversity by contrast variation, which is not easily possible for SAXS but which is quite straightforward for small-angle neutron scattering (SANS) based on selective deuteration. Here it would be interesting for example to measure the glutamate concentration in the vesicle lumen by variation of the contrast in this moiety (using deuterated glutamate). In fact, with regard to SAXS, the electron density of a glutamate solution does not differ sufficiently from the pure buffer solution to deduce the glutamate concentration. For fully activated SVs, we can estimate approximately 8000 glutamate molecules contained in the lumen (Wang etal 2019), roughly corresponding to 1500 mM. With the literature values for room temperature density of glutamate solutions, this results only in a minor increase in electron density. Note as well that even in the absence of ATP, glutamate may partition differently due to osmotic and electro-osmotic effects.

Third, and finally we may measure SVs not in a large ensemble, but in sequential high throughput mode using single particle coherent diffractive imaging (CDI) with single XFEL pulses, an approach already demonstrated for viruses (Seibert et al. 2011; Sobolev et al. 2020) and scalable to smaller biomolecular assemblies and macromolecules (Chapman 2019; Brändén et al. 2019; Oberthür 2018), which are delivered by aerosol electrospray methods (Bielecki et al. 2019). In fact, in view of the limitations outlined above, we want to advocate single-particle CDI for the important problem of SV functional dynamics. In this way, the main limitation of conventional solution SAXS due to polydispersity and loss of information by ensemble average could be overcome in a fundamental manner. Nano-focused synchrotron radiation and microfluidic sample delivery would in principle allow to serially probe smaller and potentially contamination-free SV ensembles. However, radiation damage in the highly focused beams would be prohibitive. Contrarily, radiation damage can be outrun by the ultrashort pulse length of XFEL radiation, which enable a *diffract-before-destroy* strategy. Only by both spatial and temporal photon concentration, the scattering signal can be increased without being compromised by radiation damage.

**Fig. 5:**
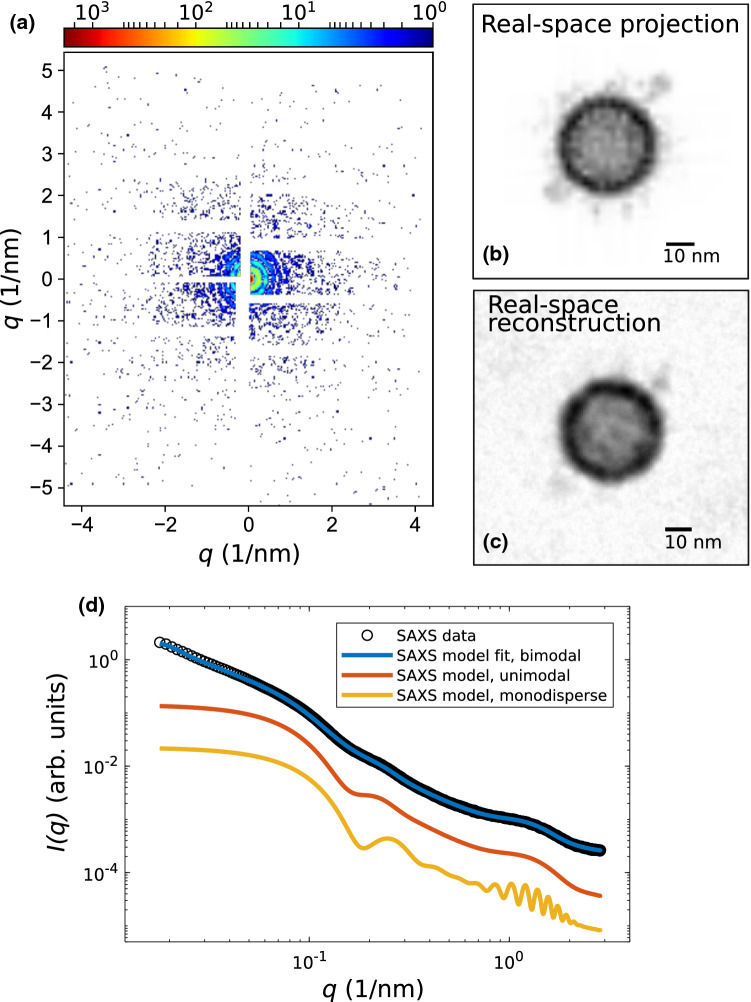
**a**–**c** Simulation results of a single-SV CDI experiment and phase reconstruction using Condor and HAWK, taking the estimated Poisson noise and the detector gaps into account. **a** Simulated single-SV diffraction pattern for the orientation of the SV shown in (**b**) using the following simulation parameters: 6 keV photon energy, 1 mJ pulse energy, 500 nm focus, AGIPD detector geometry with 4$\times$ downsampling and 0.75 m sample-to-detector distance. **b** Projected electron density of a SV based on the molecular model in (**a**). Scalebar: 10 nm. **c** Reconstruction of the real space image from (**a**) using the difference-map algorithm with 20,000 iterations, Scalebar: 10 nm. It shows that the two ATPases can be identified. **d** SAXS data (Castorph et al. 2010a) of a polydisperse ensemble of SVs with a model fit using a bimodal size distribution accounting for contaminations. This is compared to simulations with a unimodal size distribution of SVs and a monodisperse ensemble of SVs, as can be achieved by sub-ensemble averaging, in contrast to synchrotron SAXS

Figure 5 illustrated both the feasibility and information gain of a single-SV CDI experiment by a numerical simulation. The simulation of the diffraction pattern in (a) was performed by using the open-source software package *Condor* (Hantke et al. 2016), with the simulation parameters listed in the caption of Fig. 5. We have used the molecular model of a SV shown in Fig. 1a for the simulations. Details on how the molecular model of a SV is built, regarding the lipid vesicle and the composition of proteins, can be found in Takamori (2006). All atom positions are converted into a PDB file, which was used as an input file for *Condor*. Figure 5b shows the projected electron density of the SV used for the simulation, as well es the reconstruction of the real-space image from the diffraction pattern in (a). Phase reconstruction was performed by using the software package *HAWK* (Maia et al. 2010). It can be observed, that the overall shape of the SV can be quite well reconstructed, as well as the two ATPases can be identified.

However, due to the intrinsically heterogeneous nature of SVs in view of size polydispersity and to some extent of the molecular composition, a 3D reconstruction from many diffraction patterns obtained from different orientations of the SVs will be challenging and accompanied by a loss of information on the molecular level. Therefore, in addition to phase retrieval, the anisotropic SV SAXS model can be used to analyze the data in reciprocal space by using an “ in silico cleaned” monodisperse ensemble. To this end, SV diffraction patterns will be added up, following a veto-strategy to rule out images with aggregates, and radially integrated. This approach is roughly equivalent to sub-tomogram averaging in cryo-EM and will prevent loss of information by heterogeneous ensembles and thus increase structural resolution compared to conventional solution SAXS. Figure 5d shows simulated SAXS curves using the anisotropic SV SAXS model for (red) an unimodal size distribution (without contamination), and (yellow) an unimodal size distribution and monodisperse ensemble of SVs, which are compared to SAXS data obtained from SVs and the corresponding SAXS model fit including (blue) a bimodal size distribution. It can be clearly observed, that a bimodal size distribution in the first place, but also polydispersity of only an unimodal size distribution, washes out distinct features in the scattering curve, hence less structural information.

**Fig. 6:**
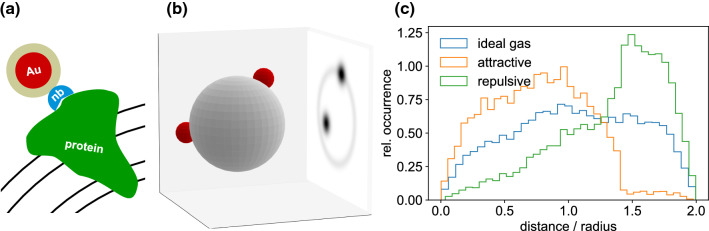
Nanobody-nanogold labeling. **a** Sketch of the gold particle attachment principle: maleimide (brown)-coated gold 1.4 nm gold particles (red) are selectively bound to desired proteins (green) such as VGLUT1 by a nanobody (blue). Black lines illustrate the lipid bilayer. **b** Schematic of an experiment probing the distribution of gold-labelled proteins (red spheres) on the SV surface. **c** From the distance histograms of the simulated 2D projections, protein interaction potentials can be inferred. Here 19,900 pairs drawn at random with a Boltzmann weight have been evaluated, for the interaction-free case (ideal gas distribution on the sphere), as well as for attractive and repulsive potentials

To gain information on the molecular level another promising approach is the use of nanobody-nanogold labels on specific proteins (Maidorn et al. 2019). Spatial proximity of proteins on the SV surface plays a role in their concerted function. Individual large proteins or protein clusters must therefore be identified in reconstructed images of individual SVs to study functional units. To achieve this goal, individual proteins can be labeled with a specific gold-nanobody probe, as illustrated in Fig. 6a. Namely, a maleimide-coated 1.4 or 5 nm gold particle is bound to a protein with a nanobody to site-selectively enhance the scattering contrast. The larger number of photons in the diffraction pattern will also help in phase retrieval and localization of the labels. In this way, we can study protein co-localization on individual SVs and unravel attractive or repulsive interaction potentials between proteins from the histogram of projected distances, see Fig. 6c. Nanobodies which specifically address single synaptic proteins, such as VGLUT1, have already been successfully expressed (Schenck et al. 2017). Of course, sample delivery is also a challenge; an aerosol injection into the vacuum of the beam path has already been developed, and is compatible even with megahertz data acquisition (Sobolev et al. 2020) at the European X-ray free electron laser. In this respect, we can expect a bright future for diffraction studies of functional states of synaptic vesicles and maybe even further synaptic organelles, yielding high throughput quantitative data potentially at very low sample consumption. The only bottleneck at this point is the still very limited beamtime available at the one or two instruments which are capable to provide the required beam and instrumental settings. Beamtime provided, a bright future may be ahead.

**Table 1:** Parameters corresponding to the least-squares fits shown in Fig. 2, comparing the SV fractions of this study to Castorph et al. (2010a). $\rho _i$ is the amplitude and $t_i$, $i \in \{in,out,tail\}$ is the width, for each of the Gaussians representing the headgroups and the tail region. $R_{g}^{i}$ and $N_{c}^{i}$, $i \in \{in,out\}$, denote the radii of gyration, and the copy numbers of the Gaussian chains, and $\sigma _c$ the corresponding electron density. All (excess) densities denote the density difference to the buffer solution. The thickness of the bilayer is $D = \sqrt{2\pi }(t_{in} + t_{tail} + t_{out})$, with $t_{in} = t_{out}$, since the bilayer is assumed to be symmetric. *R* denotes the vesicle radius defined as the center of the bilayer. $\sigma _R$ denotes the Gaussian width of the SV polydispersity, and *a* its amplitude. $R_{\mathrm {large}}$ and $\sigma _{R,\mathrm {large}}$ denote the corresponding parameters for the contamination fraction

Model fit parameter	SVs, this study	SVs, Castorph et al.	Unit
$\rho _{in}$, $\rho _{out}$	46.8	46.8	e$^{-}$ nm$^{-3}$
$\rho _{tail}$	$-28.8$	$-28.8$	e$^{-}$ nm$^{-3}$
$t_{in}$, $t_{\mathrm {out}}$	1.6	1.79	nm
$t_{tail}$	2.33	2	nm
$R_{g}^{in}$	2.51	2.86	nm
$R_{g}^{out}$	4.38	5.3	nm
$N_{c}^{in} / (4 \pi (R - D - R_{g}^{in})^2)$	0.0179	0.0084	$\mathrm {nm}^{-2}$
$N_{c}^{out} / (4 \pi (R + R_{g}^{in})^2)$	0.00136	0.0009	$\mathrm {nm}^{-2}$
$\rho _{\mathrm {c}}$	52.1	52.1	e$^{-}$ nm$^{-3}$
*R*	16.95	16.95	nm
$\sigma _{R}$	3.92	3.92	nm
$\mathrm {Amplitude}$	248.19	248.19	Arb. units
$R_{\mathrm {large}}$	277.84	328.58	nm
$\sigma _{R,\mathrm {large}}$	40.8	82.5	nm
$\mathrm {Amplitude}_{\mathrm {large}}$	0.43	1.22	Arb. unit
$\mathrm {Scale}$	1.0097	0.0838	–
$\mathrm {Constant ~ background}$	0.00109	0.00019	1/mm

**Table 2:** Fit parameters and corresponding errors ($\pm \sigma$,standard deviation) of the uptake experiments shown in Fig. 4. Parameter symbols are defined as in Table 1. In addition to the parameters of Table 1, the parameter $\rho _{\mathrm {lumen}}$ denotes the density difference of the lumen with respect to the buffer solution

Model fit parameter	SV	SV uptake control	SV uptake	Unit
$\rho _{{in}}$, $\rho _{{out}}$	31.7 (0.65)	26.53 (0.24)	31.16 (0.26)	e$^{-}$ nm$^{-3}$
$\rho _{{tail}}$	$-62.45$ (0.98)	$-67.92$ (0.16)	$-64.75$ (0.17)	e$^{-}$ nm$^{-3}$
$\rho _{\mathrm {lumen}}$	$-0.3$ (0.02)	$-0.2$ (0.05)	$-0.75$ (0.02)	e$^{-}$ nm$^{-3}$
$t_{{in}}$, $t_{{out}}$	2.47 (0.02)	2.62 (0.01)	2.5 (0.01)	nm
$t_{{tail}}$	0.65 (0.03)	0.58 (0.01)	0.7 (0.01)	nm
$R_{g}^{in}$	2.47 (0.07)	2.72 (0.01)	2.77 (0.01)	nm
$R_{g}^{out}$	5.67 (0.29)	6.58 (0.03)	6.82 (0.03)	nm
$N_{c}^{in} / (4 \pi (R - D - R_{g}^{in})^2)$	0.032 (0.002)	0.038 (0.0003)	0.044 (0.0004)	nm$^{-2}$
$N_{c}^{out} / (4 \pi (R + R_{g}^{in})^2)$	0.00102 (0.00012)	0.00081 (0.00002)	0.00107 (0.00004)	nm$^{-2}$
$\rho _{{c}}$	28.06 (1.77)	19.63 (0.18)	19.02 (0.21)	Arb. unit
*R*	16.42 (0.09)	17.56 (0.03)	18.42 (0.04)	nm
$\sigma _{R}$	4 (0.06)	3.58 (0.02)	2.14 (0.03)	nm
$\mathrm {Amplitude}$	35.64 (0.02)	35.75 (0.02)	35.51 (0.03)	Arb. units
$R_{\mathrm {large}}$	273.62 (0.47)	274.32 (0.94)	267.8 (0.42)	nm
$\sigma _{R,\mathrm {large}}$	42 (0.36)	42.88 (0.24)	39.92 (0.08)	nm
$\mathrm {Amplitude}_{\mathrm {large}}$	0.05 (0.002)	0.08 (0.001)	0.18 (0.001)	Arb. unit
$\mathrm {Scale}$	10.62 (0.15)	10.92 (0.09)	5.44 (0.04)	–
$\mathrm {Constant ~ background}$	0.0011 (0.00004)	0.0012 (0.000007)	0.0012 (0.000001)	1/mm

## Appendix A: Dynamic light scattering (DLS)

For comparison with SAXS results, and as a more accessible instrument to many research teams, dynamic light scattering (DLS) was used to measure the hydrodynamic radius of synaptic vesicles after neurotransmitter uptake. The preparation of the samples was performed directly before the measurement. SVs were stored at $-80$ $^{\circ }$C and had to be thawed on ice for about 15 min before the samples were prepared. During a measurement the remaining SVs and chemicals were kept on ice. The SVs were diluted to the desired concentration (here concentrations between 1:125 and 1:2000 were used) in an uptake buffer containing 300 mM Glycine, 5 mM HEPES, 10 mM KCl and 2 mM $\mathrm {MgSO}_4 \times 3\mathrm {H}_2\mathrm {O}$ in MilliQ. The pH was set to 7.3. Before dilution the uptake buffer was sterile filtered with $0.2\,\mu \mathrm {m}$ syringe filters (Whatman FP30/0.2 CA-S, UK). For the control measurement 10 mM K-glutamate was added, for the uptake experiment 10 mM K-glutamate and 4 mM MgATP. For the measurement $500\,\mu \mathrm {l}$ of the sample solution was filled into thoroughly cleaned cylindrical borosilicate cuvettes with a diameter of $5\,\mathrm {mm}$ (ROTILABO^®^, Roth, Germany) and sealed with a cap.

**Fig. 7:**
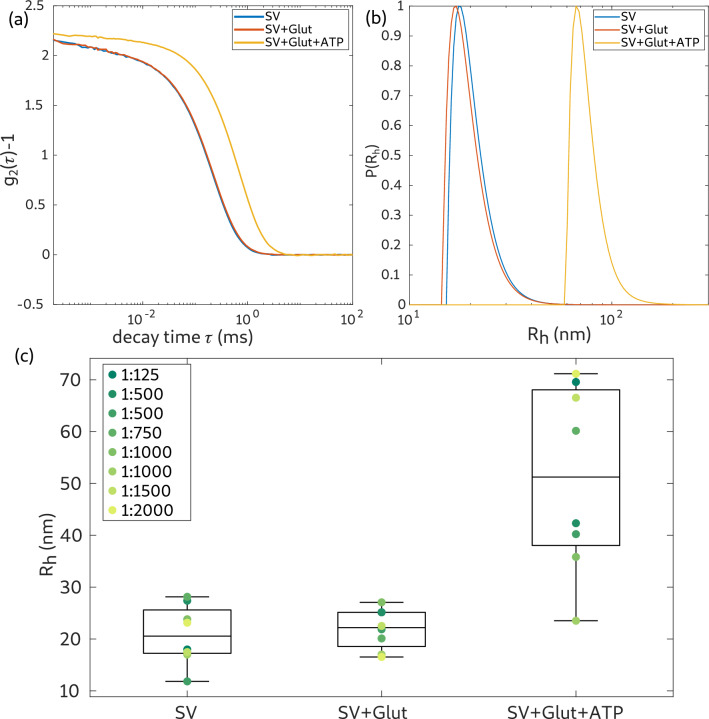
**a** Intensity correlation curves for the SV, SV control (SV+Glut) and SV uptake (SV+Glut+ATP) measurement using DLS. The vesicle concentration was 1:1500. **b** Hydrodynamic radius distribution obtained from the correlation curves in (**a**). The radii are weighted by $1/R_{\mathrm {h}}^6$ and are normalized to a maximum value of 1. **c** Distribution of the hydrodynamic radii for different vesicle concentrations. The median of the distributions is indicated by a line. For vesicle concentrations of 1:500 and 1:1000 two individual samples were measured, which are individually plotted in the graph

DLS measurements were performed using an ALV/CGS-3 Laser light scattering goniometer system (ALV GmbH, Langen, Germany) equipped with a $22\,\mathrm {mW}$ HeNe-Laser ($\lambda =632.8\,\mathrm {nm}$, UNIPHASE, model 1145P) and an ALV/LSE-5004 multiple tau correlator. The sample was placed into a heated (37 $^{\circ }$C) toluene bath. The sample was kept in this bath approximately 15 min before the measurement was started, allowing for thermal equilibration and settling of eventual dust particles. The scattered light intensity was recorded by an avalanche photo diode at a scattering angle of $90^{\circ }$ to the incident beam. Each sample was measured for 6 runs of $30\,\mathrm {s}$ and an average intensity correlation curve ($\mathrm {g}_2(\tau )-1$) was automatically calculated by the autocorrelator system. An example for such curves is shown in Fig. 7a. Based on the relationship

$$\begin{aligned} g_2(\tau )-1=\beta \Biggl |\int \limits _{0}^{\infty } p(R_h)\exp (-q^2D(R_h)\tau )\,\text {d}R\Biggl |^2 \end{aligned}$$

with coherence/contrast parameter $\beta$, momentum transfer vector *q*, diffusion constant *D* and decay time $\tau$, the distribution function of the hydrodynamic radius $\mathrm {p}(\mathrm {R}_{\mathrm {h}})$ can be obtained. Here, this was implemented based on an inverse Laplace transformation of the autocorrelation data, using the CONTIN algorithm as provided by the ALV-software (Provencher and Štêpánek 1996). The radius distribution was weighted by $1/R^6$.

The results are summarized in Fig. 7. (a) Shows the intensity correlation function ($\mathrm {g}_2(\tau )-1$) for the SV-measurement (SV) as well as the control experiment (SV+Glut) and the uptake experiment (SV+Glut+ATP) for a vesicle concentration of 1:1500. While the correlation curves of the SV and control measurement differ only by a small shift to higher $\tau$, the correlation curve of the uptake experiment exhibits a large shift, i.e. indicating a much higher relaxation time. In (b) the size distribution (weighted hydrodynamic radius) extracted from the measurements in (a) is shown. The radii of the SV measurement and the control measurement are almost identical. Contrarily, the hydrodynamic radius of the uptake experiment ($R_{\mathrm { h,uptake}}=65.5\,\mathrm {nm}$) is much larger. Similar observations (radius of the uptake experiment substantially larger) were made for all SV concentrations. However, the exact values for the radii differ substantially, without obvious correlation to the vesicle concentration. This can be concluded from a series of DLS measurements at different concentrations, see Fig. 7c, where the mean of the radius distribution for each measurement is indicated by an individual symbol and the median of these is indicated by a line. For concentrations of 1:500 and 1:1000 two samples were measured. The median of the hydrodynamic radii of the SV measurements (${\bar{R}}_{\mathrm {h,SV}}=20.56\,\mathrm {nm}$) and the control measurements (${\bar{R}}_{\mathrm {h,SV+Glut}}=22.2\,\mathrm {nm}$) differ by about $1.6\,\mathrm {nm}$, which represents a similar increase as deduced from SAXS. Note that the definition of the radius in the SAXS model refers to the bilayer center, and the (outer radius), which should be associated with the hydrodynamic radius, must be correspondingly larger, by at least half the thickness of the lipid chain region plus the headgroup thickness. Therefore, the SAXS and the DLS results for the mean SV radius are in good agreement. The DLS results are also in good agreement with earlier studies (Castorph et al. 2011). The results for the uptake experiment, however, are puzzling. The apparent radius (${\bar{R}}_{\mathrm {h,uptake}}=51.25\,\mathrm {nm}$) is twice as large after uptake than before. This unrealistically large size increase must be certainly regarded as an artifact. It could possibly be a result of aggregation, or additional relaxation times associated with other dominating modes of the active vesicle. In view of vesicle size and vesicle concentration in the sample, a clustering of vesicles, resulting in slowing down, an apparent increase of vesicle size seems unlikely, but the relaxation times are certainly due to a similar indirect effect, not to single vesicle diffusion. This underlines the necessity to probe the structural effects by a structural probe such as diffraction or imaging, rather than indirectly via dynamics.

## Appendix B: alternative fitting strategy

**Fig. 8:**
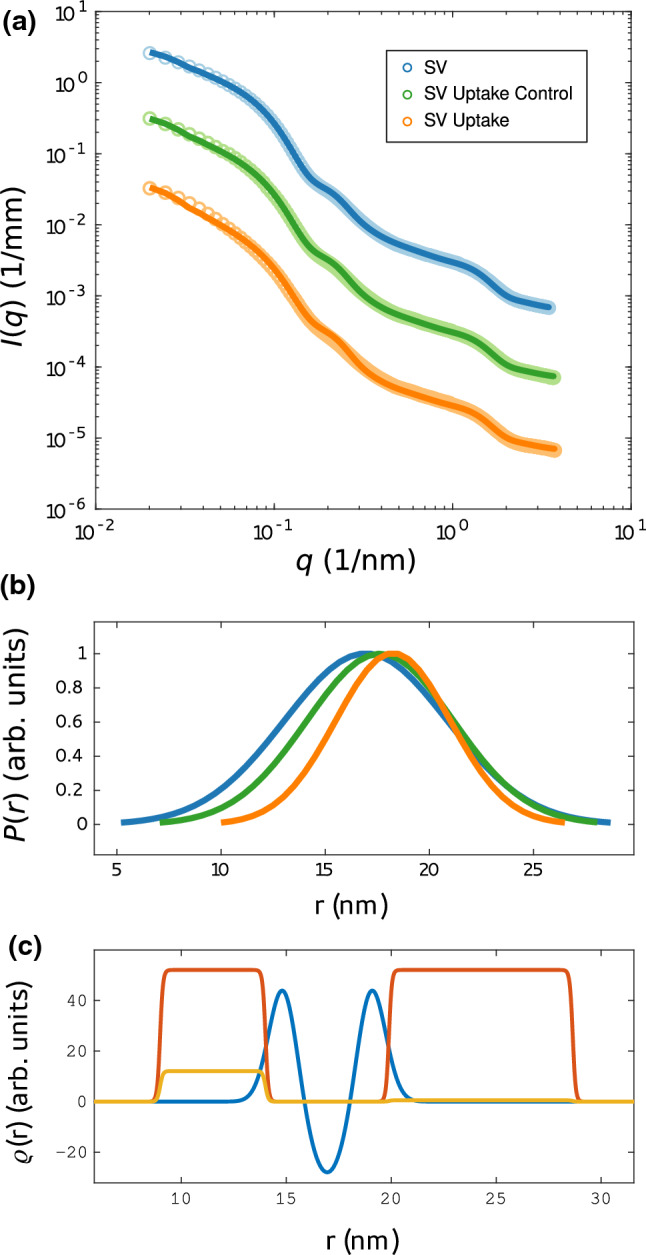
Structural changes of SVs after neurotransmitter uptake. **a** SAXS data and anisotropic-SV model fits for SV only (blue, $\chi _{\mathrm {red}}^{2} = 68.8$), SV uptake control (green, $\chi _{\mathrm {red}}^{2} = 277.6$), and SV uptake (orange, $\chi _{\mathrm {red}}^{2} = 770.3$). **b** Normalized Gaussian size distributions obtained from least-square fits shown in (**a**) with the corresponding colors. The size distribution of large particles was kept constant for each fit. **c** Electron density profile (EDP) obtained from the least-squares fit of SV only. The structural parameters of the EDP were kept constant for the fits of SV uptake control and SV uptake. The EDP of the lipid bilayer is shown in blue, the Gaussian chains *local* in red and the Gaussian chains *averaged* in yellow

Figure 8 and Table 3 present results of an alternative fitting strategy, where the electron density of the EDP components, namely protein, lipid headgroup, and lipid tail were kept fixed, while bilayer thickness, protein number density and radius of gyration, and both size distributions were freely varied for the SV reference. Next, for the SV uptake control and SV uptake data, only the size distribution of the small fraction was free, and all other parameters were kept fixed. No density contrast of the lumen was taken into account, i.e. $\rho _{\mathrm {lumen}}=0$. This setting was tested to rule out that the observed size increase is simply an artifact resulting from over-parameterisation.The fits show that the data can still fairly be modeled by only the size increase, as the dominant effect causing the changes in the SAXS signal, see Fig.8. However, $\chi _{\mathrm {red}}^{2}$-values are much higher than in the freely varied case, indicating that the changes in the EDP are also significant.

**Table 3:** Parameters corresponding to the least-squares fits shown in Fig. 8, presenting an alternative fitting strategy of the uptake experiments, with fixed EDP

Model fit parameter	SV	SV uptake control	SV uptake	Unit
$\rho _{{in}}$, $\rho _{{out}}$	46.8	46.8	46.8	e$^{-}$ nm$^{-3}$
$\rho _{{tail}}$	$-28.8$	$-28.8$	$-28.8$	e$^{-}$ nm$^{-3}$
$\sigma _{{in}}$, $\sigma _{{out}}$	0.68	0.68	0.68	nm
$\sigma _{{tail}}$	0.99	0.99	0.99	nm
$R_{g}^{in}$	2.51	2.51	2.51	nm
$R_{g}^{out}$	4.38	4.38	4.38	nm
$N_{c}^{in}/(4 \pi (R - D - R_{g}^{in})^2)$	0.0179	0.0179	0.0179	nm$^{-2}$
$N_{c}^{out} / (4 \pi (R + R_{g}^{in})^2)$	0.00136	0.00136	0.00136	nm$^{-2}$
$\rho _{{c}}$	52.1	52.1	52.1	e$^{-}$ nm$^{-3}$
*R*	16.95	17.64	18.25	nm
$\sigma _{R}$	3.92	3.53	2.75	nm
$\mathrm {Amplitude}$	248.19	156.52	85.44	Arb. units
$R_{\mathrm {large}}$	277.84	277.84	277.84	nm
$\sigma _{R,\mathrm {large}}$	40.8	40.8	40.8	nm
$\mathrm {Amplitude}_{\mathrm {large}}$	0.43	0.43	0.43	Arb. unit
$\mathrm {Scale}$	1.0097	1.3379	1.6731	–
$\mathrm {Constant ~ background}$	0.00109	0.00121	0.00117	1/mm

## Appendix C: covariance matrix

From the $N=100$ independent realisations (see methods above), the covariance matrix was determined for all three samples of the uptake experiment, as shown below. See Tables 4, 5, 6 and 7.

**Table 4:** Explanation of fit parameters $P_{i}$

$P_{1}$	$\rho _{{in}}$, $\rho _{{out}}$
$P_{2}$	$\rho _{{tail}}$
$P_{3}$	$D_{1/2}$, half thickness of the shell
$P_{4}$	*R*
$P_{5}$	$N_{c}^{out} / (4 \pi (R + R_{g}^{in})^2)$
$P_{6}$	$R_{g}^{out}$
$P_{7}$	$\rho _{{c}}$
$P_{8}$	$N_{c}^{in} / (4 \pi (R - D - R_{g}^{in})^2)$
$P_{9}$	Scale
$P_{10}$	Constant background
$P_{11}$	$\sigma _{R}$
$P_{12}$	$\mathrm {Amplitude}$
$P_{13}$	$R_{g}^{out}$
$P_{14}$	Fraction $t_{\mathrm {headgroup}}/D$
$P_{15}$	$R_{\mathrm {large}}$
$P_{16}$	$\mathrm {Amplitude}_{\mathrm {large}}$
$P_{17}$	$\sigma _{R,\mathrm {large}}$
$P_{18}$	$\rho _{\mathrm {lumen}}$

**Table 5:** Correlation matrix for the fit parameters *P* obtained for SV only

	$P_{1}$	$P_{2}$	$P_{3}$	$P_{4}$	$P_{5}$	$P_{6}$	$P_{7}$	$P_{8}$	$P_{9}$	$P_{10}$	$P_{11}$	$P_{12}$	$P_{13}$	$P_{14}$	$P_{15}$	$P_{16}$	$P_{17}$	$P_{18}$
$P_{1}$	1	0.8	$-0.96$	$-0.5$	0.55	$-0.45$	0.56	$-0.13$	$-0.98$	$-0.15$	$-0.38$	$-0.97$	$-0.52$	$-0.99$	$-0.62$	0.4	$-0.52$	$-0.74$
$P_{2}$	0.8	1	$-0.91$	$-0.89$	0.93	$-0.89$	0.94	$-0.7$	$-0.88$	$-0.71$	$-0.86$	$-0.88$	$-0.92$	$-0.72$	$-0.88$	0.87	$-0.83$	$-0.52$
$P_{3}$	$-0.96$	$-0.91$	1	0.67	$-0.72$	0.64	$-0.73$	0.34	0.97	0.38	0.58	0.97	0.7	0.93	0.76	$-0.6$	0.67	0.68
$P_{4}$	$-0.5$	$-0.89$	0.67	1	$-0.99$	0.97	$-0.96$	0.86	0.59	0.9	0.96	0.6	0.98	0.39	0.87	$-0.96$	0.84	0.35
$P_{5}$	0.55	0.93	$-0.72$	$-0.99$	1	$-0.99$	0.99	$-0.88$	$-0.65$	$-0.9$	$-0.98$	$-0.66$	$-0.99$	$-0.44$	$-0.88$	0.98	$-0.87$	$-0.34$
$P_{6}$	$-0.45$	$-0.89$	0.64	0.97	$-0.99$	1	$-0.99$	0.93	0.57	0.95	0.99	0.59	0.99	0.34	0.85	$-0.99$	0.86	0.24
$P_{7}$	0.56	0.94	$-0.73$	$-0.96$	0.99	$-0.99$	1	$-0.89$	$-0.67$	$-0.9$	$-0.98$	$-0.69$	$-0.99$	$-0.46$	$-0.87$	0.98	$-0.86$	$-0.31$
$P_{8}$	$-0.13$	$-0.7$	0.34	0.86	$-0.88$	0.93	$-0.89$	1	0.28	0.98	0.95	0.3	0.88	0.02	0.69	$-0.95$	0.74	$-0.03$
$P_{9}$	$-0.98$	$-0.88$	0.97	0.59	$-0.65$	0.57	$-0.67$	0.28	1	0.29	0.51	0.99	0.62	0.96	0.68	$-0.52$	0.61	0.67
$P_{10}$	$-0.15$	$-0.71$	0.38	0.9	$-0.9$	0.95	$-0.9$	0.98	0.29	1	0.96	0.31	0.92	0.04	0.73	$-0.96$	0.77	0.02
$P_{11}$	$-0.38$	$-0.86$	0.58	0.96	$-0.98$	0.99	$-0.98$	0.95	0.51	0.96	1	0.53	0.98	0.27	0.82	$-0.99$	0.84	0.18
$P_{12}$	$-0.97$	$-0.88$	0.97	0.6	$-0.66$	0.59	$-0.69$	0.3	0.99	0.31	0.53	1	0.64	0.95	0.69	$-0.54$	0.62	0.67
$P_{13}$	$-0.52$	$-0.92$	0.7	0.98	$-0.99$	0.99	$-0.99$	0.88	0.62	0.92	0.98	0.64	1	0.41	0.87	$-0.98$	0.86	0.3
$P_{14}$	$-0.99$	$-0.72$	0.93	0.39	$-0.44$	0.34	$-0.46$	0.02	0.96	0.04	0.27	0.95	0.41	1	0.54	$-0.29$	0.43	0.74
$P_{15}$	$-0.62$	$-0.88$	0.76	0.87	$-0.88$	0.85	$-0.87$	0.69	0.69	0.73	0.83	0.69	0.87	0.54	1	$-0.84$	0.8	0.47
$P_{16}$	0.4	0.87	$-0.6$	$-0.96$	0.98	$-0.99$	0.98	$-0.95$	$-0.52$	$-0.96$	$-0.99$	$-0.54$	$-0.98$	$-0.29$	$-0.84$	1	$-0.85$	$-0.17$
$P_{17}$	$-0.52$	$-0.83$	0.67	0.84	$-0.87$	0.86	$-0.86$	0.74	0.61	0.76	0.84	0.63	0.86	0.43	0.8	$-0.85$	1	0.34
$P_{18}$	$-0.74$	$-0.52$	0.68	0.35	$-0.34$	0.24	$-0.31$	$-0.03$	0.67	0.02	0.18	0.66	0.3	0.74	0.47	$-0.17$	0.34	1

**Table 6:** Correlation matrix for the fit parameters *P* obtained for SV Uptake

	$P_{1}$	$P_{2}$	$P_{3}$	$P_{4}$	$P_{5}$	$P_{6}$	$P_{7}$	$P_{8}$	$P_{9}$	$P_{10}$	$P_{11}$	$P_{12}$	$P_{13}$	$P_{14}$	$P_{15}$	$P_{16}$	$P_{17}$	$P_{18}$
$P_{1}$	1	0.95	$-0.9$	$-0.15$	0.62	$-0.62$	0.82	0.01	$-0.94$	0.44	$-0.36$	$-0.17$	$-0.45$	$-0.98$	$-0.2$	0.44	0.06	$-0.38$
$P_{2}$	0.95	1	$-0.85$	$-0.19$	0.69	$-0.74$	0.88	$-0.08$	$-0.92$	0.31	$-0.51$	$-0.35$	$-0.54$	$-0.93$	$-0.24$	0.56	0.03	$-0.42$
$P_{3}$	$-0.9$	$-0.85$	1	0.01	$-0.58$	0.56	$-0.68$	$-0.26$	0.85	$-0.34$	0.24	0.16	0.49	0.93	0	$-0.37$	0.06	0.17
$P_{4}$	$-0.15$	$-0.19$	0.01	1	$-0.70$	0.48	$-0.26$	0.11	$-0.05$	0.23	0.18	$-0.01$	0.6	0.02	0.51	$-0.2$	$-0.28$	0.41
$P_{5}$	0.62	0.69	$-0.58$	$-0.7$	1	$-0.91$	0.77	$-0.03$	$-0.54$	$-0.14$	$-0.55$	$-0.12$	$-0.91$	$-0.53$	$-0.36$	0.67	0.05	$-0.4$
$P_{6}$	$-0.62$	$-0.74$	0.56	0.48	$-0.91$	1	$-0.91$	0.27	0.65	0.22	0.71	0.14	0.82	0.53	0.28	$-0.83$	0.01	0.36
$P_{7}$	0.82	0.88	$-0.68$	$-0.26$	0.77	$-0.91$	1	$-0.36$	$-0.87$	0.03	$-0.7$	$-0.17$	$-0.62$	$-0.74$	$-0.24$	0.78	0.01	$-0.38$
$P_{8}$	0.01	$-0.08$	$-0.26$	0.11	$-0.03$	0.27	$-0.36$	1	0.1	0.32	0.45	0.02	$-0.22$	$-0.12$	0.15	$-0.35$	$-0.11$	0.16
$P_{9}$	$-0.94$	$-0.92$	0.85	$-0.05$	$-0.54$	0.65	$-0.87$	0.1	1	$-0.38$	0.49	0.12	0.41	0.93	0.06	$-0.59$	0.05	0.25
$P_{10}$	0.44	0.31	$-0.34$	0.23	$-0.14$	0.22	0.03	0.32	$-0.38$	1	0.25	$-0.12$	0.17	$-0.5$	0.04	$-0.27$	0.07	$-0.05$
$P_{11}$	$-0.36$	$-0.51$	0.24	0.18	$-0.55$	0.71	$-0.7$	0.45	0.49	0.25	1	0.17	0.49	0.28	0.40	$-0.9$	0.08	0.48
$P_{12}$	$-0.17$	$-0.35$	0.16	$-0.01$	$-0.12$	0.14	$-0.16$	0.02	0.12	$-0.12$	0.17	1	0.09	0.21	0.03	$-0.02$	0.05	0.08
$P_{13}$	$-0.45$	$-0.54$	0.49	0.6	$-0.91$	0.82	$-0.62$	$-0.22$	0.4	0.17	0.49	0.09	1	0.39	0.25	$-0.64$	0.02	0.26
$P_{14}$	$-0.98$	$-0.93$	0.93	0.02	$-0.53$	0.53	$-0.74$	$-0.12$	0.93	$-0.5$	0.28	0.21	0.39	1	0.12	$-0.36$	$-0.02$	0.31
$P_{15}$	$-0.2$	$-0.24$	0	0.51	$-0.36$	0.28	$-0.25$	0.15	0.06	0.04	0.41	0.04	0.25	0.12	1	$-0.3$	$-0.39$	0.94
$P_{16}$	0.44	0.56	$-0.37$	$-0.2$	0.67	$-0.83$	0.79	$-0.35$	$-0.59$	$-0.27$	$-0.9$	$-0.02$	$-0.64$	$-0.36$	$-0.3$	1	$-0.07$	$-0.34$
$P_{17}$	0.06	0.03	0.06	$-0.28$	0.05	0.01	0.01	$-0.11$	0.05	0.07	0.07	0.05	0.02	$-0.02$	$-0.39$	$-0.07$	1	$-0.33$
$P_{18}$	$-0.38$	$-0.42$	0.17	0.41	$-0.4$	0.36	$-0.38$	0.16	0.25	$-0.05$	0.48	0.08	0.26	0.31	0.94	$-0.34$	$-0.33$	1

**Table 7:** Correlation matrix for the fit parameters *P* obtained for SV Uptake Control

	$P_{1}$	$P_{2}$	$P_{3}$	$P_{4}$	$P_{5}$	$P_{6}$	$P_{7}$	$P_{8}$	$P_{9}$	$P_{10}$	$P_{11}$	$P_{12}$	$P_{13}$	$P_{14}$	$P_{15}$	$P_{16}$	$P_{17}$	$P_{18}$
$P_{1}$	1	0.99	$-0.83$	$-0.33$	0.61	$-0.65$	0.89	0.33	$-0.97$	0.76	$-0.24$	$-0.97$	$-0.54$	$-0.99$	$-0.28$	0.33	0.07	$-0.31$
$P_{2}$	0.99	1	$-0.84$	$-0.28$	0.56	$-0.67$	0.95	0.22	$-0.98$	0.73	$-0.18$	$-0.99$	$-0.53$	$-0.98$	$-0.19$	0.34	$-0.01$	$-0.23$
$P_{3}$	$-0.83$	$-0.84$	1	0.12	$-0.43$	0.53	$-0.77$	$-0.34$	0.83	$-0.58$	$-0.02$	0.82	0.54	0.87	0.02	$-0.16$	0.05	0.01
$P_{4}$	$-0.33$	$-0.28$	0.12	1	$-0.78$	0.64	$-0.24$	$-0.3$	0.14	$-0.09$	0.32	0.16	0.62	0.24	0.54	$-0.37$	$-0.23$	0.52
$P_{5}$	0.61	0.56	$-0.43$	$-0.78$	1	$-0.9$	0.5	0.55	$-0.46$	0.23	$-0.7$	$-0.47$	$-0.81$	$-0.53$	$-0.73$	0.73	0.43	$-0.78$
$P_{6}$	$-0.65$	$-0.67$	0.53	0.64	$-0.9$	1	$-0.73$	$-0.28$	0.57	$-0.19$	0.55	0.59	0.79	0.59	0.54	$-0.75$	$-0.23$	0.56
$P_{7}$	0.89	0.95	$-0.77$	$-0.24$	0.5	$-0.73$	1	0.01	$-0.92$	0.58	$-0.12$	$-0.93$	$-0.51$	$-0.88$	$-0.1$	0.38	$-0.11$	$-0.11$
$P_{8}$	0.33	0.22	$-0.34$	$-0.3$	0.55	$-0.28$	0.01	1	$-0.21$	0.32	$-0.44$	$-0.21$	$-0.66$	$-0.34$	$-0.46$	0.24	0.44	$-0.53$
$P_{9}$	$-0.97$	$-0.98$	0.83	0.14	$-0.46$	0.57	$-0.92$	$-0.21$	1	$-0.78$	0.15	0.99	0.44	0.98	0.12	$-0.28$	0.03	0.17
$P_{10}$	0.76	0.73	$-0.58$	$-0.09$	0.23	$-0.19$	0.58	0.32	$-0.78$	1	0.04	$-0.77$	$-0.26$	$-0.78$	$-0.01$	$-0.06$	$-0.08$	$-0.07$
$P_{11}$	$-0.24$	$-0.18$	$-0.02$	0.32	$-0.7$	0.55	$-0.12$	$-0.44$	0.15	0.04	1	0.16	0.37	0.17	0.82	$-0.82$	$-0.62$	0.9
$P_{12}$	$-0.97$	$-0.99$	0.82	0.16	$-0.47$	0.59	$-0.93$	$-0.21$	0.99	$-0.77$	0.16	1	0.45	0.98	0.14	$-0.3$	0.02	0.19
$P_{13}$	$-0.54$	$-0.53$	0.54	0.62	$-0.81$	0.79	$-0.51$	$-0.66$	0.44	$-0.26$	0.37	0.45	1	0.51	0.36	$-0.49$	$-0.18$	0.38
$P_{14}$	$-0.99$	$-0.98$	0.87	0.24	$-0.53$	0.59	$-0.88$	$-0.34$	0.98	$-0.78$	0.17	0.98	0.51	1	0.2	$-0.27$	$-0.04$	0.24
$P_{15}$	$-0.28$	$-0.19$	0.02	0.54	$-0.73$	0.54	$-0.1$	$-0.46$	0.12	$-0.01$	0.82	0.14	0.36	0.2	1	$-0.78$	$-0.63$	0.92
$P_{16}$	0.33	0.34	$-0.16$	$-0.37$	0.73	$-0.75$	0.38	0.24	$-0.28$	$-0.06$	$-0.82$	$-0.3$	$-0.49$	$-0.27$	$-0.78$	1	0.42	$-0.71$
$P_{17}$	0.07	$-0.01$	0.05	$-0.23$	0.43	$-0.23$	$-0.11$	0.44	0.03	$-0.08$	$-0.62$	0.02	$-0.18$	$-0.04$	$-0.63$	0.42	1	$-0.67$
$P_{18}$	$-0.31$	$-0.23$	0.01	0.52	$-0.78$	0.56	$-0.11$	$-0.53$	0.17	$-0.07$	0.9	0.19	0.38	0.24	0.92	$-0.71$	$-0.67$	1
